# Predicting the performance of automated crystallographic model-building pipelines

**DOI:** 10.1107/S2059798321010500

**Published:** 2021-11-29

**Authors:** Emad Alharbi, Paul Bond, Radu Calinescu, Kevin Cowtan

**Affiliations:** a Department of Computer Science, University of York, Heslington, York YO10 5GH, United Kingdom; b Department of Information Technology, University of Tabuk, Tabuk, Saudi Arabia; c Department of Chemistry, University of York, Heslington, York YO10 5DD, United Kingdom

**Keywords:** structure solution, model building, software, prediction, automated pipelines

## Abstract

Proteins are macromolecules that perform essential biological functions which depend on their three-dimensional structure. Determining this structure involves complex laboratory and computational work. For the computational work, multiple software pipelines have been developed to build models of the protein structure from crystallographic data. Each of these pipelines performs differently depending on the characteristics of the electron-density map received as input. Identifying the best pipeline to use for a protein structure is difficult, as the pipeline performance differs significantly from one protein structure to another. As such, researchers often select pipelines that do not produce the best possible protein models from the available data. Here, a software tool is introduced which predicts key quality measures of the protein structures that a range of pipelines would generate if supplied with a given crystallographic data set. These measures are crystallographic quality-of-fit indicators based on included and withheld observations, and structure completeness. Extensive experiments carried out using over 2500 data sets show that the tool yields accurate predictions for both experimental phasing data sets (at resolutions between 1.2 and 4.0 Å) and molecular-replacement data sets (at resolutions between 1.0 and 3.5 Å). The tool can therefore provide a recommendation to the user concerning the pipelines that should be run in order to proceed most efficiently to a depositable model.

## Abstract 2

A machine-learning model was used to predict the performance of four crystallographic model-building pipelines (*ARP*/*wARP*, *Buccaneer*, *Phenix AutoBuild* and *SHELXE*) and their combinations.

## 1. Introduction

The first protein structures were determined in the 1950s using X-ray crystallography (Kendrew *et al.*, 1958 ▸). By 2020, the number of solved protein structures deposited in the Protein Data Bank (PDB) exceeded 154 000 (Berman *et al.*, 2000 ▸; https://www.rcsb.org/stats/summary). To enable this progress, researchers have automated the computational work of determining the protein structure from X-ray crystallographic data sets. Multiple protein model-building pipelines have been developed within the last three decades: *ARP*/*wARP* (Perrakis *et al.*, 1999 ▸; Lamzin & Wilson, 1993 ▸; Morris *et al.*, 2003 ▸; Langer *et al.*, 2008 ▸, 2013 ▸), *Buccaneer* (Cowtan, 2006 ▸, 2008 ▸), *Phenix AutoBuild* (Terwilliger *et al.*, 2008 ▸; Liebschner *et al.*, 2019 ▸) and *SHELXE* (Sheldrick, 2008 ▸, 2010 ▸; Thorn & Sheldrick, 2013 ▸; Usón & Sheldrick, 2018 ▸). In recent studies, we have shown that the performance of these pipelines differs significantly from one protein structure to another (Alharbi *et al.*, 2019 ▸), which makes selecting a particular pipeline difficult, and that using a pair of pipelines is sometimes the best option (Alharbi *et al.*, 2020 ▸), which greatly increases the number of options that crystallographers can choose from.

An important step in building the protein structure involves solving the phase problem. The phase problem may be solved using either molecular replacement or experimental phasing methods; see, for example, McCoy & Read (2010 ▸) and Evans & McCoy (2008 ▸). These methods lead to electron-density maps with rather different properties: in the case of experimental phasing the maps usually contain noise due to ambiguity in the experimental phasing, whereas in the molecular-replacement case errors in the map can arise from possible bias towards the molecular-replacement model. The resolution of the experimental observations, the quality of experimental phasing or the similarity of the molecular-replacement model, and many other features such as ice rings may also affect the quality of the data. Each of these factors impact the performance of different model-building algorithms in different ways (Vollmar *et al.*, 2020 ▸; Alharbi *et al.*, 2019 ▸; Morris *et al.*, 2004 ▸).

The model-building process also contains stochastic elements. The placement of the first atom or residue in a chain will in turn influence the placement of all subsequent elements, and so substantially different model-building results may be obtained from very slight perturbations of the initial conditions. This is addressed in one model-building pipeline by building multiple models at each stage of the process (Terwilliger *et al.*, 2008 ▸).

**Figure 1:**
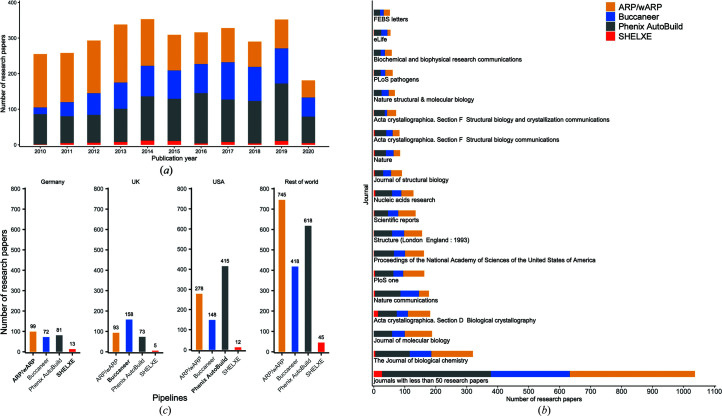
Analysis of the crystallographic model-building pipelines used in 3273 PDB protein-structure research papers published between 2010 and 2020. The papers were identified using either their PubMed identifier or DOI obtained from the PDB. We omitted research papers that used multiple pipelines. We compared the number of uses of each pipeline in its base country, depending on the home country of the first author’s organization. (*a*) The number of research papers by publication year for each pipeline. (*b*) The journals in which the research papers were published; journals with fewer than 50 research papers are combined into one group. (*c*) The number of uses of each pipeline in its base country and across the rest of the world; the pipeline names are shown in bold in their base-country plot.

We examined a selection of 3273 research papers cited in the PDB to evaluate how crystallographers currently choose which model-building software pipeline to use, by searching for occurrences of the pipeline names in the text of each paper and excluding papers where the search results were ambiguous or where multiple tools were mentioned. The results are plotted against year, journal and the country of the first author in Fig. 1 ▸. The most striking feature of this analysis is the correlation between the first author’s country and the country where each pipeline has been developed, with US researchers more likely to use *Phenix Autobuild*, UK researchers more likely to use *Buccaneer* and German researchers more likely to use *ARP*/*wARP*. While there are practical reasons which might explain this correlation (for example access to developers and workshops), it would be surprising if cognitive biases such as affinity bias (Ashforth & Mael, 1989 ▸), to which we are all subject, did not play a role.

To help to eliminate this bias, we have developed a software tool that uses a machine-learning (ML) model to predict the performance of a wide range of model-building pipelines and pipeline combinations for a given crystallographic data set. Our prediction tool serves three purposes.

(i) To provide users with a more efficient route to a higher-quality depositable structure for their specific data set.
(ii) To challenge users to try different pipelines, and multiple combinations of pipelines, on the basis of likely performance rather than on the basis of familiarity or affinity to the pipeline developers. Given that all pipelines provide very convenient user interfaces, the overhead of trying a new pipeline will cost less than the effort of model completion from a suboptimal starting point.
(iii) To assist future developers in the development of meta-tools which make use of multiple pipelines to further automate the process of structure solution and to obtain more complete models.

To the best of our knowledge, this is the first ML solution that guides the user in selection of the model-building pipelines that are most suitable for a given crystallographic data set. While a predictive model that employs similar ML techniques was recently proposed by Vollmar *et al.* (2020 ▸), that model addresses the complementary problem of predicting the usefulness of collected crystallographic data sets.

## 2. Predictive model

### 2.1. Data sets

We used data sets from three sources to train and evaluate our ML predictive model: 1203 experimental phasing data sets from the Joint Center for Structural Genomics (JCSG; van den Bedem *et al.*, 2011 ▸; Alharbi *et al.*, 2019 ▸), 32 newer experimental phasing data sets deposited between 2015 and 2021 and taken from the PDB, and 1332 molecular-replacement (MR) data sets from Bond *et al.* (2020 ▸). These data sets correspond to two techniques that can be used to build a protein structure. Experimental phasing is when the phases are determined from the observed data using the features of special atoms, such as a large number of electrons; see, for example, Dauter & Dauter (2017 ▸). In contrast, MR obtains initial phases from a known protein structure that is similar to the protein structure that we want to build; see, for example, Evans & McCoy (2008 ▸).

The resolution of the JCSG experimental phasing data sets ranges from 1.2 to 4.0 Å, with the low-resolution data sets augmented by simulation as in Alharbi *et al.* (2019 ▸), the resolution of the PDB experimental phasing data sets ranges from 1.1 to 5.8 Å, and the resolution of the MR data sets ranges from 1.0 to 3.5 Å. Lower resolution data sets have fewer experimental observations, which decreases the performance of the protein-building pipelines.

The way in which we partitioned these data sets into data for training and data for evaluation of our ML model is described in Section 2.5.

### 2.2. Crystallographic model-building pipelines

The four pipeline versions used in our work are *Phenix AutoBuild* version 1.14, *Buccaneer* in *CCP*4*i* version 7.0.066, *ARP*/*wARP* version 8 and *SHELXE* version 2019/1. These pipelines were run using the default parameters, both individually and in pairwise combinations where the protein model produced by a first pipeline *x* was supplied as input to a second pipeline *y*.

### 2.3. Protein structure evaluation

We focused on predicting three protein structure evaluation measures, namely *R*_free_, *R*_work_ and structure completeness. *R*_free_ and *R*_work_ measure the fit of the protein structure against the observed data, with *R*_free_ only using observations which are not used in the refinement calculation: typically 5% of the data (Brünger, 1992 ▸). Structure completeness is the percentage of residues in the deposited protein model with a matching residue in the built model. Residues are considered to match if they have the same type and the distance between their C^α^ atoms is less than 1 Å.

### 2.4. Electron-density map features

We trained our ML prediction model using the resolution of the crystallographic data set and the following measures of the quality of the electron-density map as input features.

(i) R.m.s.d.: the root-mean-square deviation of the electron density from the mean of the map.
(ii) Skew: the third moment of the electron density about the mean, which measures the asymmetry of the electron-density histogram (Terwilliger *et al.*, 2009 ▸).
(iii) Maximum density: the highest density of the electron-density map.
(iv) Minimum density: the lowest density of the electron-density map.
(v) Sequence identity: the sequence identity calculated by superposition of the homologue chain onto the target chain using *GESAMT* (Krissinel, 2012 ▸; Bond *et al.*, 2020 ▸).

### 2.5. Predictive model training

The individual pipelines were run on all data sets listed in Section 2.1. The pipeline combinations were only run on the experimental phasing data sets, as building protein models from such ‘raw data’ can often be improved by using combinations of pipelines (Alharbi *et al.*, 2020 ▸). The results of these runs are described in detail in our recent work (Alharbi *et al.*, 2019 ▸, 2020 ▸). The data sets and the protein structures obtained from these runs were used to train and evaluate the predictive ML model as follows.

(i) 80% of the JCSG experimental phasing data sets and 80% of the MR data sets were used to train the predictive model.
(ii) The remaining 20% of the JCSG experimental phasing and MR data sets, and all 32 PDB experimental phasing data sets, were used to evaluate the trained model.

We used random forests (Breiman, 2001 ▸) as implemented in the Weka framework (Hall *et al.*, 2009 ▸; Frank *et al.*, 2016 ▸) for the predictive model, as this approach showed the lowest error rate across the ML algorithms that we tested, which included a support vector machine (Cortes & Vapnik, 1995 ▸) and the *RepTree* decision-tree algorithm. We varied the number of trees in the random forest from 1 to 5000 in geometric sequence, and 1024 was chosen for the final training as this showed the lowest error rate. The depth of the trees was set to unlimited, and bagging (Breiman, 1996 ▸) was used to reduce the variance. We trained the predictive model using a 173-node high-performance cluster with 7024 Intel Xeon Gold/Platinum cores and a total memory of 42 TB.

**Figure 2:**
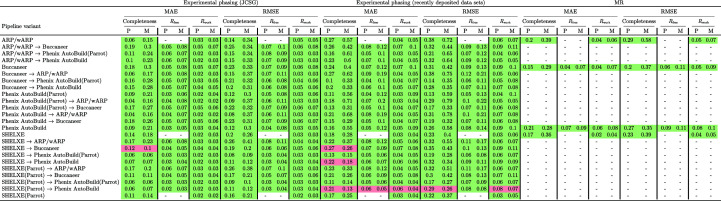
Mean absolute error (MAE) and root-mean-squared error (RMSE) of structure completeness and *R*_free_/*R*_work_ for two types of experimental phasing data sets and for molecular-replacement (MR) data sets. *ARP*/*wARP* and *SHELXE* are not used for *R*_free_. For the MR data sets, only individual pipelines were run. MAE and RMSE were calculated for the ML predictive model (P) and median predictor (M) used as a baseline (*Zero-R*) model.

A separate regression ML model (random forest model) was trained for each of the 24 pipeline variants (*i.e.* individual pipelines or pipeline combinations) in Fig. 2 ▸ and for each of the three structure evaluation measures in Section 2.3 relevant to the considered pipeline variant. For instance, *R*_free_ is not relevant for *ARP*/*wARP* and *SHELXE* with and without *Parrot* used on their own, so no ML model was built for these individual pipelines and *R*_free_. We obtained a total of 69 and ten regression ML models for experimental phasing and for MR, respectively. Our predictive model consists of these regression ML models taken together.

We used the root-mean-square error (RMSE) and mean absolute error (MAE) measures to compare the accuracy of our predictive model with that of a ‘baseline’ predictive model. In line with the standard practice for the evaluation of regression models, we used the *Zero-R* algorithm as a baseline predictive model (Choudhary & Gianey, 2017 ▸). Given a pipeline variant and any evaluation data set, the *Zero-R* algorithm predicts that the *R*_free_/*R*_work_ and structure completeness for the structure built by the pipeline would be the same as the median *R*_free_/*R*_work_ and structure completeness for the training data sets, respectively.

To evaluate the accuracy of the predictive model for data sets of different resolutions, we partitioned the evaluation data sets into classes based on their resolutions, and we examined the prediction errors for each such class. Finally, to show the time saved by running only the pipeline variant predicted to build the best protein structure for a data set, we compared the execution time of this pipeline with the time required to run all of the pipeline variants for that data set.

To quantify the uncertainty of the ML prediction, we calculated prediction intervals using the kernel estimator method from Frank & Bouckaert (2009 ▸). The width of these intervals reflects the prediction uncertainty. As such, we sort and report the pipelines in increasing prediction interval width order, with pipelines of similar prediction uncertainty (*i.e.* with no more than 5% difference in prediction interval width) grouped together.

Finally, we generate a script for each pipeline and pipeline combination, ensuring that the users of our tool can run the individual pipelines and pipeline combinations in the manner used to obtain the training data sets for our ML prediction model. Furthermore, these ready-to-run scripts are customized based on data provided by the tool users.

## 3. Predictive model evaluation

### 3.1. Evaluation of the crystallographic data-set features used for model training

**Figure 3:**
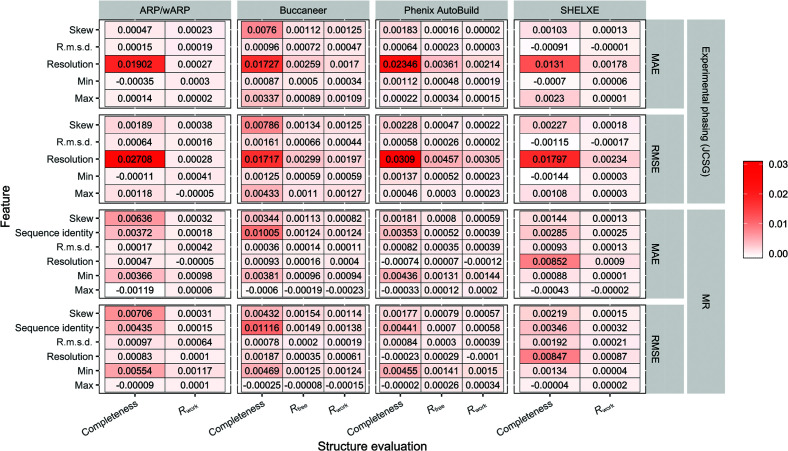
Ablation studies showing the difference in MAE and RMSE when the ML model was trained on all features and when one feature is removed at a time. Higher values indicate more important features.

We evaluated the importance of the features used to train our predictive model by removing one feature at a time and comparing the accuracy of the model trained without that feature with the accuracy of the predictive model when trained on all of the features. Fig. 3 ▸ shows the difference in MAE and RMSE when one feature is removed compared with when all of the features are used in training for each of the four individual pipelines, with separate MAE and RMSE presented for the JCSG experimental phasing and MR data sets.

This analysis indicates that *Phenix AutoBuild* and *ARP*/*wARP* are more dependent on the data-set resolution than *Buccaneer*, which is in line with previous results (Alharbi *et al.*, 2019 ▸). However, *Phenix AutoBuild* and *ARP*/*wARP* are less sensitive to the resolution for MR data sets compared with experimental phasing data sets. R.m.s.d. and skew have different effects on the performance of the pipelines. For example, *Buccaneer* is affected by these two features more than *Phenix AutoBuild* for the experimental phasing data set, indicating a greater dependence on the noise level in the starting map. For MR data sets, the sequence identity affected the performance of all pipelines, with the highest effect for *Buccaneer*.

### 3.2. Evaluation of predictive model performance

Fig. 2 ▸ shows the MAE and RMSE for both types of data sets (experimental phasing and MR) for each of the three protein structure evaluation measures. For the JCSG experimental phasing data sets, both the MAE (0.04–0.19) and RMSE (0.08–0.26) for predicting the protein structure completeness are higher than the MAE and RMSE for the other measures. The values of MAE (0.02–0.06) and RMSE (0.02–0.08) decreased when predicting *R*_free_/*R*_work_. For MR data sets, the MAE of structure completeness increased to 0.15–0.21 and the RMSE to 0.20–0.29. The MAE of *R*_free_/*R*_work_ was between 0.02 and 0.07, compared with the RMSE, which is between 0.04 and 0.09.

Different levels of predictability were achieved for different pipeline variants. For the experimental phasing data sets and *ARP*/*wARP* after *Phenix AutoBuild*, the predictive model achieved the lowest MAE for structure completeness (0.04), with a similar RMSE, which indicates a small number of large error predictions. On the other hand, for MR data sets, the MAE for structure completeness for *ARP*/*wARP* and *Phenix AutoBuild* run individually increased to 0.20 and 0.21, respectively. *Buccaneer* run individually and after *ARP*/*wARP* or *Phenix AutoBuild* showed the lowest predictability, with MAE and RMSE values above 0.17.

*R*_free_/*R*_work_ are more predictable across all pipeline variants and for both types of data sets, with lower MAE and RMSE values than those achieved for structure completeness. For the JCSG experimental phasing data sets, the predictive model achieved a low MAE for *R*_work_ (0.02–0.03) and only a slightly larger MAE for *R*_free_ (0.03–0.05) for all of the individual pipelines. The MAE obtained for pipeline combinations and *R*_work_ ranged between 0.02 and 0.05, and that for *R*_free_ varied between 0.04 and 0.06. RMSE is slightly higher than MAE for both the individual and the combined pipelines. For the MR data sets, the MAE of *R*_work_ is between 0.02 and 0.06, with the lowest value being obtained for *SHELXE*, and the MAE for *R*_free_ is between 0.04 and 0.07. Finally, the RMSEs of *R*_free_ and *R*_work_ are between 0.06 and 0.09 and between 0.04 and 0.08, respectively.

**Figure 4:**
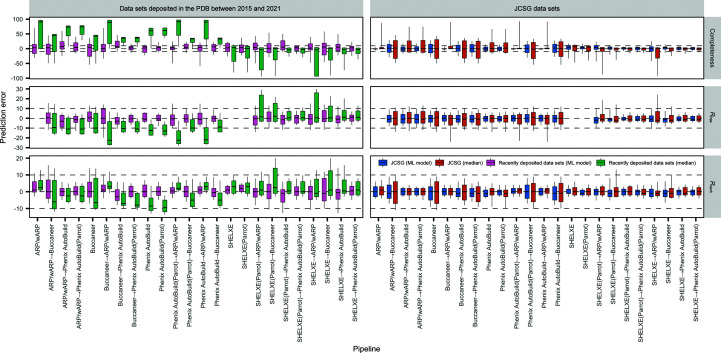
Prediction error for the ML predictive model and the median predictor for recently deposited and JCSG experimental phasing data sets.

Compared with the baseline *Zero-R* predictive model (see Section 2.5), our predictive model achieved lower or much lower MAE and RMSE prediction errors for almost all of the pipeline variants, types of data sets and protein structure evaluation measures, *i.e.* for 288 of the 296 entries in Fig. 2 ▸. Notably, the predictions for recently PDB-deposited experimental phasing data sets (which we did not use in the training of the predictive model) also have a much lower error for our predictive model than for the *Zero-R* predictive model (Fig. 4 ▸), with the exception of the predictions for *SHELXE* before *Buccaneer* and *Phenix AutoBuild*, for which the *Zero-R* baseline model predictions achieve similar or marginally lower errors.

**Figure 5:**
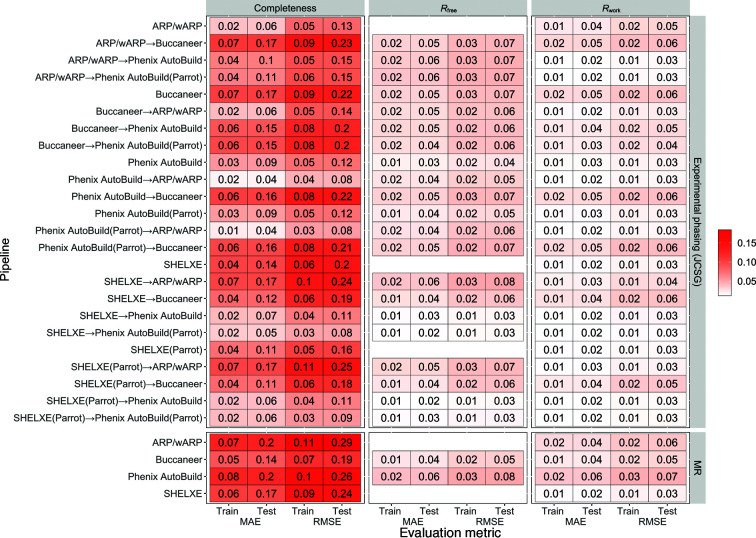
MAE and RMSE of structure completeness and *R*_free_/*R*_work_ for training and testing for the JCSG experimental phasing data sets and the MR data sets. The entries are shaded based on the magnitude of the difference in MAE and RMSE between the training and testing data sets.

To evaluate the fitting of our predictive model, Fig. 5 ▸ shows the difference in MAE and RMSE between training and testing for the JCSG experimental phasing and the MR data sets. The difference in MAE and RMSE between training and testing data sets for structure completeness is higher than that in *R*_work_/*R*_free_ for the JCSG experimental phasing and the MR data sets. When comparing the pipelines by structure completeness, *Phenix AutoBuild* and *Buccaneer* have the lowest error difference for the JCSG experimental phasing and the MR data sets, respectively. For *R*_work_/*R*_free_, the pipelines have a smaller difference in MAE and RMSE between the training and testing data sets compared with the structure completeness.

**Figure 6:**
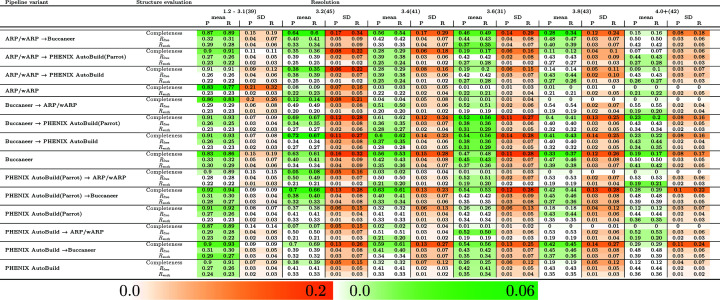
Mean and standard deviation (SD) of the real and predicted structure evaluation measures for the JCSG experimental phasing data sets grouped based on resolution, with the number of data sets in each group shown in parentheses. The entries are shaded based on the magnitude of the difference between the real (R) and predicted (P) results.

To further evaluate the accuracy of our predictive model, we analysed the mean and standard deviation (SD) of the predicted and actual protein structure evaluation measures for the crystallographic data sets grouped based on their resolutions. Figs. 6 ▸ and 7 ▸ show the results of this analysis for JCSG experimental phasing data sets for the pipeline variants without *SHELXE* and with *SHELXE*, respectively. For resolutions between 1.2 and 3.1 Å, the predicted and actual mean and SD values are very close for most pipeline variants. The spread of the predicted structure completeness for *ARP*/*wARP* run alone and run after *SHELXE* has a higher SD compared with the completeness achieved when the pipelines were run in reality. At worse than 3.2 Å, the predicted *R*_free_/*R*_work_ have mean and SD values close to the real results, while the predicted structure completeness has a larger difference in the SD and a smaller difference in the mean than the actual results.

**Figure 8:**
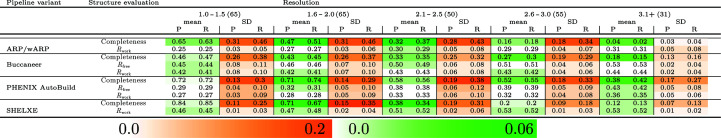
Mean and SD of the real and predicted structure evaluation measures for the MR data sets grouped based on resolution, with the number of data sets in each group shown in parentheses. The entries are shaded based on the difference between the real (R) and predicted (P) results.

Fig. 8 ▸ shows the results of the same analysis as above for the MR data sets. The mean of all the predicted structure evaluation measures as well as the SD values for the predicted *R*_free_/*R*_work_ are close to the actual results. However, at resolutions better than 3.0 Å the difference between the SD for the predicted and actual structure completeness is larger than that for *R*_free_/*R*_work_. At resolutions of 3.1 Å or worse, this difference decreases significantly.

To evaluate the predictive model uncertainty, we grouped the pipelines using the method described in Section 2.5. We evaluated this by checking whether the pipeline with the lowest prediction error was classified in the first group for each protein structure in our testing data set. For the JCSG experimental phasing data set, 85%, 94% and 91% of the pipelines with the lowest prediction error were classified in the first group for structure completeness, *R*_free_ and *R*_work_, respectively. For the MR data set the percentages were 60%, 69% and 87%, respectively.

**Figure 9:**
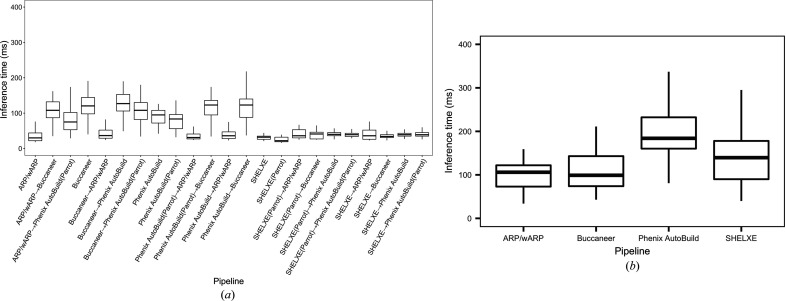
Inference time for the predictive model for individual pipelines and pipeline combinations. For each data set in the JCSG experimental phasing and MR data sets, the inference time is the total time taken to predict the structure completeness, *R*_free_ and *R*_work_. (*a*) Inference time for the JCSG experimental phasing data sets and (*b*) inference tine for the MR data sets.

Fig. 9 ▸ shows the inference time of the predictive model for individual pipelines and pipeline combinations for the JCSG experimental phasing and MR data sets. The inference time is the total time taken to predict the structure completeness and *R*_free_/*R*_work_. The *SHELXE* variants for the JCSG experimental phasing data set and *ARP*/*wARP* and *Buccaneer* for the MR data set have the lowest inference times.

### 3.3. Evaluation of the recommended pipeline variant

To further evaluate our predictive model, we analysed the potential benefits of using the pipeline variant recommended by the model, *i.e.* the pipeline variant predicted to achieve the best completeness or *R*_free_/*R*_work_ for each of the data sets.

**Figure 10:**
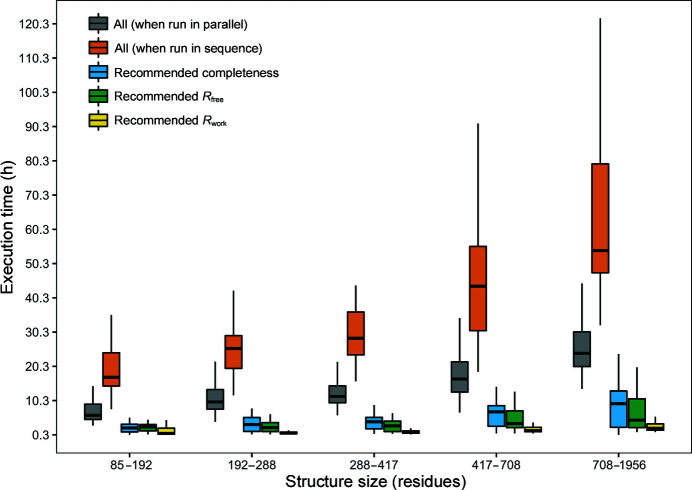
Execution time required to run all of the pipeline variants (in parallel and in sequence) versus the execution time required to run the pipeline recommended by the predictive model (for best completeness, best *R*_free_ and best *R*_work_) for the JCSG experimental phasing data sets.

To this end, we first analysed the time savings that can be achieved by using the recommended pipeline variant instead of running all of the pipeline variants in order to obtain the best possible structure. Fig. 10 ▸ shows the total execution time when running all of the pipeline variants and when only the pipeline recommended by our predictive model was run. The time saved (on the powerful high-performance cluster mentioned in Section 2.5) was up to 20 h for a small protein structure and up to 60 h for large structures. When these pipeline variants were ran in parallel on our high-performance cluster, this time saving was reduced; however, running the recommended pipeline still saved up to 30 h when building large structures.

**Figure 11:**
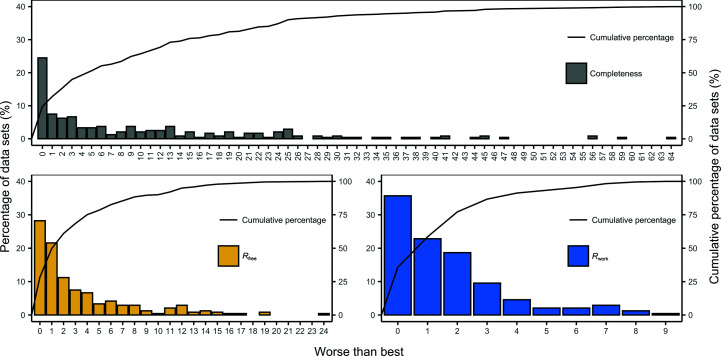
Difference between the best completeness, *R*_free_ and *R*_work_ achieved by running all of the pipeline variants and running the recommended pipeline variant for the JCSG experimental phasing data sets. The percentage of the data sets for each difference group is shown on the left and the cumulative percentage is shown on the right.

Next, we analysed how close the completeness and *R*_free_/*R*_work_ of the protein structure built by the recommended pipeline variant was to the best completeness and *R*_free_/*R*_work_ values achievable by running all of the pipeline variants. Figs. 11 ▸ and 12 ▸ present the results of this analysis for the JCSG experimental phasing and MR data sets, respectively. These results show that the recommended pipeline variant built protein structures with a completeness, *R*_free_ and *R*_work_ within only 1% of those of the best pipeline for 32%, 50% and 59% of the JCSG experimental phasing data sets and 70%, 99% and 71% of the MR data sets, respectively, and within only 5% of those of the best pipeline for 52%, 78% and 93% of the JCSG experimental phasing data sets and 83%, 100% and 87% of the MR data sets, respectively.

Finally, for each of the 15 research papers that we could find for our testing MR data sets that mentioned the pipeline used to build the protein structure, we compared the pipeline used in the paper with the pipeline variant recommended by our predictive model. To ensure a fair comparison, we ran the pipeline used in the paper and the pipeline recommended by our predictive model using the same search model to obtain initial phases for each structure. This search model could not be the same as that used for the PDB-deposited structure, which is unavailable.

**Figure 13:**
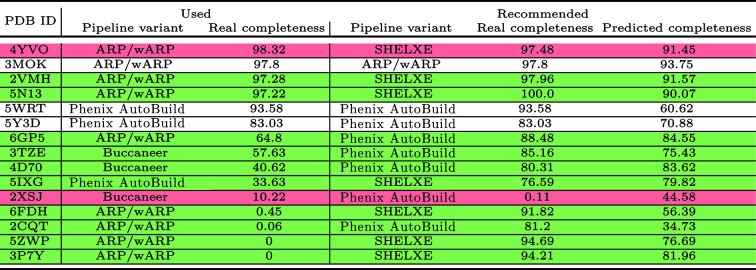
Real structure completeness achieved by the pipeline that was used to solve the protein structure when deposited in the PDB and by the pipeline recommended by the predictive model for the MR data sets.

Fig. 13 ▸ presents the structure completeness achieved by the pipeline that was chosen to solve the protein structure when deposited in the PDB compared with the completeness achieved by our recommended pipeline for each of these MR data sets. As shown in this figure, our recommended pipeline achieved better completeness than the other pipeline for ten of the 15 protein structures, and an identical completeness for three additional structures for which the predictive model recommended the same pipeline as that used to build the PDB structure. The recommended pipeline achieved worse completeness for only two of the 15 protein structures (with a decrease in completeness of less than 1% for one of these).

## 4. Discussion

We have presented a predictive model of the performance of four widely used protein model-building pipelines and of their pairwise combinations. We have separately trained this predictive model for both experimental phasing and molecular-replacement data sets and for three commonly used structure evaluation measures. Using this predictive model, we aim to help users choose the best pipeline for solving their protein structure based on the features of their starting data, to encourage them to use pipelines which may be less familiar to them and to increase the joint use of multiple pipelines, as doing so is likely to yield a more complete and more refined structure.

The features were calculated in scale-dependent measures; however, scale-independent measures are more natural in the crystallographic context. The scale-dependent measures were implemented first, yielding almost indistinguishable results. We assume that this is due to the machine-learning model effectively factoring out scale internally.

The MAE and RMSE analysis showed that *R*_free_ and *R*_work_ are more predictable than structure completeness in both experimental phasing and MR data sets. This unpredictability differs between the pipeline variants, suggesting that the electron-density map features have different effects on the performance of the pipelines. The predictability of pipelines involving *Phenix Autobuild* tends to be higher, which is likely to be due to the use of multiple models to offset stochastic effects. Both the MAE and RMSE for our predictive model are significantly lower than the MAE and RMSE for the training data set median used by the baseline, *Zero-R* predictive model.

When comparing the individual data sets by using the mean and SD for the real and predicted structure evaluation measures at high resolution, which is considered to be an easier case, the performance of the pipelines is more predictable than at low resolution. When the data sets become worse in terms of resolution (which typically also means that the phases become worse), the difference in SD between the real and predicted results becomes larger.

The pipeline variant predicted to build the best protein structure frequently produced structures with the same or similar completeness and/or *R*_free_/*R*_work_ as the best pipeline variant. Moreover, using the pipeline variant recommended by our predictive model save days of pipeline execution time on high-specification computers, and the time saved increases when the protein structure is larger. Finally, the predictive model can be used to try massive search models in MR cases, enabling the selection of good initial phases (Simpkin *et al.*, 2018 ▸; Bibby *et al.*, 2012 ▸).

Future work will consider a multi-task method for predicting structure completeness, *R*_free_ and *R*_work_, and will combine the ML models into a single model. We envisage that this could lead to more accurate predictions and to better pipeline ranking. Moreover, we will explore additional ML algorithms, for example *XGBoost* (Chen & Guestrin, 2016 ▸), as this may improve our predictive model.

## 5. Availability

We implemented the predictive model described in the paper as a web application that is publicly available and free to use at http://www.robin-predictor.org. The source code for the application is available at https://doi.org/10.15124/ee9d169f-c34b-44f2-8c75-3b68e7cd68a8. 

## Figures and Tables

**Figure 7:**
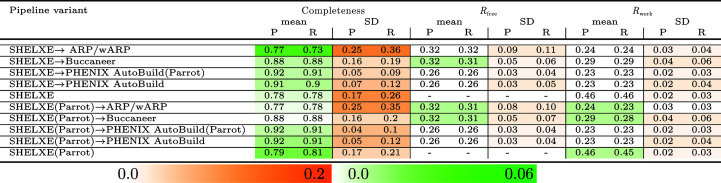
Mean and SD of the real and predicted structure evaluation measures for the JCSG experimental phasing data sets for *SHELXE* and its combinations. The resolutions of the data sets are between 1.2 and 3.1 Å. The results are shaded based on the difference between the real (R) and predicted (P) results.

**Figure 12:**
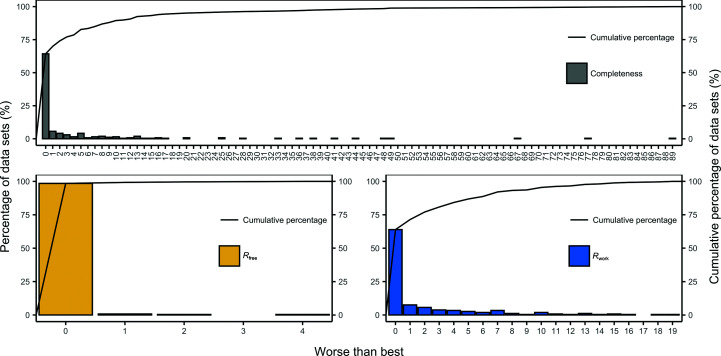
Difference between the best completeness, *R*_free_ and *R*_work_ achieved by running all of the pipeline variants and running the recommended pipeline variant for the MR data sets. The percentage of the data sets for each difference group is shown on the left and the cumulative percentage is shown on the right
